# Correlation of cardiac troponin T and APACHE III score with all-cause in-hospital mortality in critically ill patients with acute pulmonary embolism

**DOI:** 10.1515/med-2022-0534

**Published:** 2022-08-01

**Authors:** Hongxia Wang, Yang Ji, Keke Zhang, Guangqiang Shao

**Affiliations:** Respiratory and Critical Care Medicine Department, The University of Hong Kong-Shenzhen Hospital, 1, Haiyuan 1st Road, Futian District, Shenzhen, Guangdong, People’s Republic of China; Respiratory and Critical Care Medicine Department, The University of Hong Kong-Shenzhen Hospital, Shenzhen, China; Respiratory and Critical Care Medicine Department, The University of Hong Kong-Shenzhen Hospital, Shenzhen, China; Department of Surgery, Division of Thoracic Surgery, The University of Hong Kong Shenzhen Hospital, Shenzhen, China

**Keywords:** acute physiologic and chronic health evaluation, biomarker, cardiac troponin T, mortality, pulmonary embolism, ROC

## Abstract

Pulmonary embolism (PE) is a fatal condition particularly in critically ill patients. We determined the association among the cardiac troponin T (cTnT) level, acute physiologic and chronic health evaluation (APACHE III) scoring system, and in-hospital mortality in critically ill patients with acute PE. A total of 501 patients with PE were initially enrolled. According to the multivariable logistic regression model for in-hospital mortality, the odds ratio of the cTnT level and APACHE III score was 1.96 (95% confidence interval [CI] = 1.18–3.24, *P* = 0.008) and 1.03 (95% CI = 1.02–1.05, *P* < 0.001), respectively. The area under the curve (AUC) of cTnT and APACHE III score for in-hospital mortality was 0.630 (95% CI = 0.586–0.672, *P* = 0.03) and 0.740 (95% CI = 0.699–0.778, *P* = 0.02), respectively. The discriminatory cTnT and APACHE III threshold values for in-hospital mortality were 0.08 ng/L and 38 score, respectively; the sensitivities and specificities of cTnT were 46.48 and 83.10%, respectively, whereas those of the APACHE III score were 74.88 and 54.19%, respectively. The cTnT and APACHE III scores were combined in the logistic analysis model, and a regression equation was derived to calculate the in-hospital mortality. The AUC was found to increase to 0.788 (95% CI = 0.734–0.840, *P* = 0.025). The sensitivity and specificity increased to 84.5 and 71.4%, respectively. The cTnT and APACHE III scores exhibited a significant association with in-hospital mortality of critically ill patients with PE. In conclusion, these parameters in combination can significantly improve the in-hospital mortality prediction.

## 1 Introduction

Pulmonary embolism (PE) is a fatal disease having an annual incidence of 39–115 cases per 100 thousand people [1]. The mortality rate of patients with untreated PE is 30% [2], whereas those of high-risk and moderate-risk patients with PE are 25–52% [3] and 8–15% [4], respectively. Mortality can be reduced by identifying high-risk patients through simple and easily available methods and by administering treatment in a timely manner. Predicting in-hospital mortality in critically ill patients with acute PE is challenging, and even the simplified PE severity index (sPESI), the most common tool for determining the prognosis and severity of PE, is considered unsuitable for mortality prediction in these patients. sPESI is calculated based on the following indicators: sex (10 points for men and 0 point for women), heart rate >110 beats/min (20 points), tumor (30 points), heart failure (10 points), chronic lung disease (10 points), systolic blood pressure <100 mmHg (30 points), respiratory rate <30 beats/min (20 points), body temperature <36°C (20 points), mental change (60 points), and arterial blood gas analysis oxygen saturation <90% (20 points). Generally, sPESI indicators have only two grades, which aggravates the disease severity level among critically ill patients to such an extent that makes the stratification of critically ill patients with acute PE challenging. Therefore, identifying more specific and comprehensive tools to stratify these critically ill patients is essential.

Another most extensively used system to evaluate the disease severity and prognosis of patients in the intensive care unit (ICU) is the acute physiologic and chronic health evaluation (APACHE III) scoring system. This scoring system was developed and expanded based on the APACHE II scoring system, and it comprises age, physiological scores, and health status as indicators. The APACHE III scoring system has been used in previous studies to predict hospital mortality in patients undergoing surgery [5] and in those with liver cirrhosis [6] and respiratory failure [7]; however, none of the studies have investigated its efficacy in predicting mortality of patients with acute PE.

Troponin, a cardiac disease marker, is a specific and sensitive marker for myocardial infarction. An increased troponin level was found to be significantly associated with right ventricular dysfunction [8], which is a crucial mortality predictor in acute PE [9]. However, the usefulness of troponin in predicting the prognosis of acute PE remains to be confirmed. A study by Jiménez et al. demonstrated that the elevated troponin level could not predict mortality adequately [10]. Furthermore, a study by El-Menyar et al. indicated that the increased troponin level is significantly related to high mortality in PE patients [11].

Although the APACHE III score can be a mortality predictor in patients in the ICU and cardiac troponin T (cTnT) can predict the mortality of patients with PE, no study has investigated the role of both APACHE III and cTnT as the predictor for in-hospital mortality in patients with acute PE admitted in the ICU. The current study explored the relationship between the cTnT level and APACHE III score and the all-cause in-hospital mortality of such patients. The study also attempted to determine whether all-cause in-hospital mortality prediction could be improved by the combined use of these parameters in these special study populations.

## 2 Materials and methods

### 2.1 Patient and public involvement

Neither patients nor the public were involved in the design, conduct, reporting, or dissemination of this research.

### 2.2 Ethics approval statement

This study was exempted from the institutional review board review and informed consent from patients was not required because an anonymous public database was used to retrieve data, thus waiving the need for ethical approval.

### 2.3 Database

The present retrospective study used the data extracted from the Medical Information Mart for Intensive Care III (MIMIC-III), which is an extensive, free access database containing de-identified health-related data collected from nearly 40,000 patients admitted to the ICU in the Beth Israel Deaconess Medical Center from 2001 to 2012. Informed consent was not required because the current study used data from a third-party anonymous public database, and our institution is exempted from institutional review board approval. A researcher certified by the National Institute of Health online training course (certification number: 39067458) extracted data by using PostgreSQL Tools (v. 4.24).

### 2.4 Study population and data extraction

**Figure 1:**
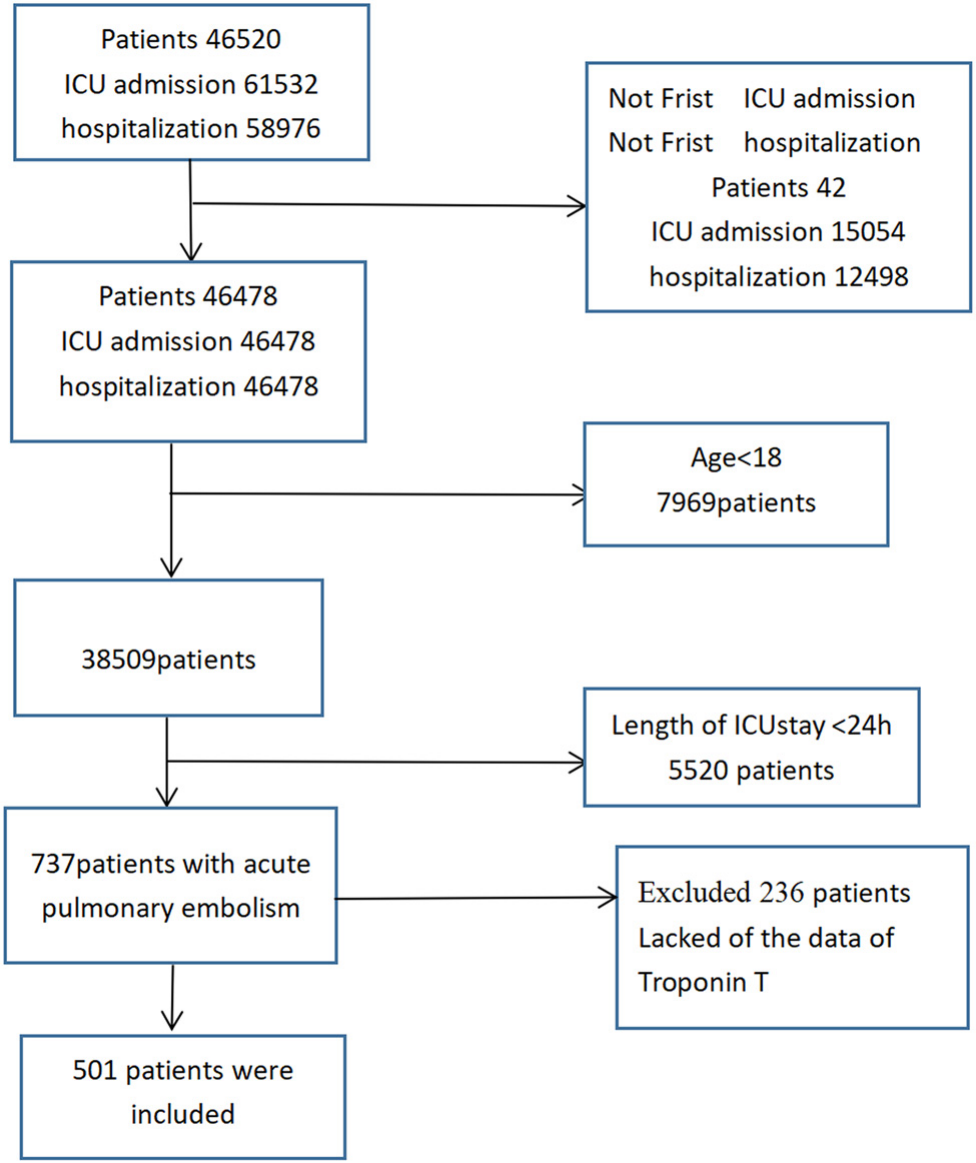
Flow chart depicting the patient selection process. Abbreviations: ICU, intensive care unit.

Patients with an age of more than 18 years who were admitted to the ICU due to acute PE and stayed in the ICU for more than 24 h were recruited as study participants. Patients diagnosed as having acute PE during their ICU stay were not included in the study, and those with unavailable data for the cTnT level were also excluded. The first ICU admission record in the database of patients with more than one record was utilized. Figure 1 summarizes the detailed procedure for participant selection.

The SQL program was used to extract the following clinical data: PE was recognized according to ICD-9-CM codes (41,519); demographic information included age, sex, weight, height, sequential organ failure assessment (SOFA), Glasgow coma score (GCS), systemic inflammatory response syndrome (SIRS), simplified acute physiology score (SAPS), and treatment variables such as renal replacement therapy and mechanical ventilation (MV). Information on comorbidities such as bacterial pneumonia, ventilator-associated pneumonia, chronic obstructive pulmonary disease, cardiac dysrhythmias, congestive heart failure, hypertension, uncomplicated and complicated diabetes, bacterial pneumonia, malignant tumor, and deep vein thrombosis, which were also determined according to ICD-9-CM codes, was extracted. Laboratory measurements comprised white blood cell (WBC) count, platelet count, hemoglobin level, cTnT level, serum creatinine, potassium (K^+^) and sodium (Na^+^) levels, blood urea nitrogen levels, and partial pressure of carbon dioxide (PCO_2_) and oxygen (PO_2_).

### 2.5 Definitions

In the database, cTnT values <0.01 were input as 0.009, whereas cTnT values >25 were input as 25.1, but the proportion of total were not >5%. “cTnT max” denotes the maximum value during hospitalization, whereas “cTnT min” denotes the minimum value during hospitalization.

#### 2.5.1 APACHE III scoring system

The APACHE Ⅲ scoring system is a critical illness evaluation method proposed by Knaus in 1991. It includes the acute physiology score (APS), chronic health status score (CHS), and age score. The APS score ranges from 0 to 252, the CHS score ranges from 4 to 23, and the age score ranges from 0 to 24; the total score ranges from 0 to 299. The APS includes 17 parameters of the Apache III physiological scoring system: heart rate, mean arterial pressure, body temperature, respiratory rate, arterial oxygen partial pressure, alveolar–arferial oxygen partial pressure (only for the patients with intubation), hematocrit, leukocyte count, creatinine, 24 h urine volume, urea nitrogen, serum sodium, albumin, total bilirubin, blood glucose, acid–base imbalance score and GCS. The CHS includes liver failure, lymphoma, tumor metastasis, leukemia, immunosuppressive, and cirrhosis.

### 2.6 Study outcome

In-hospital mortality after ICU admission was the major outcome of this study.

### 2.7 Statistical analyses

Data are presented as the mean value and standard deviation, numbers (percentages), or median (25% quartile, 75% quartile) according to the distribution and type of variables. The Kruskal–Wallis test and Chi-square (or Fisher’s exact) test were employed to compare group differences. A logistic regression model with a stepwise backward elimination method was constructed to analyze the all-cause in-hospital mortality. We input all the variables in Table 1 in the logistic regression model. The variables with a significance level of 0.05 were entered in the final multivariable logistic regression model. A variance inflation factor (VIF) was used to test potential multicollinearity, with a value of ≥5 indicating multicollinearity and the VIF of the final model was 4.7. The variables cTnT level, APACHE III score, age, BMI, hemoglobin level, WBC count, invasive MV, and malignant tumor were entered in the final model (Table 2). Age, hemoglobin, WBC count, invasive MV, and malignant tumor were also the components of the APACHE III scoring system. In order to facilitate clinical application, we simplified the model and selected a combination of only the cTnT level and APACHE III score to estimate the in-hospital mortality. The ROC curve was drawn, and the corresponding area under the curve (AUC), sensitivity, specificity, and optimal threshold value were calculated. All statistical tests were two-sided and conducted using Stata software (version 15.0). A *P* value of <0.05 was considered statistically significant.

**Table 1:** Comparison of clinical characteristics and laboratory findings between survivors and non-survivors

	Total (*n* = 501)	Survivors (*n* = 430)	Non-survivors (*n* = 71)	*P*-value
Age (years)	65.63 ± 16.3	64.3 ± 16.5	73.29 ± 12.6	<0.001
Males	265 (52.89%)	234 (54.42%)	31 (43.66%)	0.09
Females	236 (47.11%)	196 (45.58%)	40 (56.34%)	
BMI	25.0 (20.5, 27.9)	25.4 (20.8, 28.3)	22.4 (18.6, 25.0)	<0.001
Score				
SAPS	17 (13, 20)	16.3 (13, 20)	21.6 (17, 25)	<0.001
SIRS	2.9 (2, 4)	2.8 (2, 4)	3.2 (3, 4)	<0.001
GCS	13.7 (14, 15)	13.9 (14, 15)	12.8 (13, 15)	0.15
SOFA	3.87 (1, 6)	3.51 (1, 5)	6.02 (3, 9)	<0.001
APACHE III	43.5 (30, 52)	40.6 (28, 49)	60.5 (40, 73)	<0.001
MV				
Invasive	196 (39.12%)	152 (35.35%)	44 (61.97%)	<0.001
NIV	194 (9.8%)	170 (10.3%)	24 (7.6%)	0.89
Sepsis	151 (30.14%)	126 (29.3%)	25 (35.2%)	0.31
Renal replacement therapy on first day	21 (4.19%)	19 (4.42%)	2(2.82%)	0.53
Comorbidities				
DVT	142 (28.3%)	124 (28.8%)	18 (25.4%)	0.54
Bacterial pneumonia	9 (1.8%)	8 (1.86%)	1 (1.41%)	0.79
Ventilator-associated Pneumonia	17 (3.39%)	16 (3.72%)	1 (1.41%)	0.31
COPD	90 (17.96%)	74 (17.21%)	16 (22.54%)	0.27
Coronary	71 (14.17%)	62 (14.42%)	9 (12.68%)	0.69
Cardiac dysrhythmias	62 (12.38%)	56 (13.02%)	6 (8.45%)	0.27
Acute myocardial infarction	16 (3.20%)	15 (3.49%)	1 (1.41%)	0.35
Congestive heart failure	167 (33.3%)	148 (34.4%)	19 (26.7%)	0.20
Complicated diabetes	28 (5.59%)	25 (5.81%)	3 (4.23%)	0.58
Uncomplicated diabetes	112 (22.36%)	101 (23.49%)	11 (15.49%)	0.13
Hypertension	235 (46.91%)	201 (46.74%)	34 (47.89%)	0.06
Malignant tumor	132 (26.35%)	106 (24.65%)	26 (36.6%)	0.03
Liver disease	19 (3.79%)	18 (4.19%)	1 (1.41%)	0.23
Cerebral infarction	21 (4.19%)	19 (4.42%)	2 (2.28%)	0.53
Vitals first day				
Heartrate, min	91 (79, 103)	91 (80, 103)	91 (76, 105)	0.85
SBP, mmHg	117 (106, 126)	118 (107, 127)	112 (101, 124)	0.012
DBP, mmHg	63 (55, 70)	64 (56, 70)	60 (53, 65)	<0.001
Resprate, min	21 (17, 24)	21 (17, 24)	22 (18, 25)	0.10
Temp, °C	36.8 (36.3, 37.2)	36.8 (36.4, 37.2)	36.6 (36.3, 37.1)	0.29
SpO_2_	97 (95, 98)	97 (95, 98)	96 (94, 98)	0.19
Laboratory parameters				
cTnT initial, ng/mL	0.15 (0.01, 0.1)	0.11 (0.01, 0.09)	0.33 (0.01, 0.27)	<0.001
cTnT max ng/mL	0.32 (0.01, 0.2)	0.30 (0.01, 0.16)	0.52 (0.04, 0.42)	<0.001
cTnT min ng/mL	0.07 (0.01, 0.05)	0.06 (0.01, 0.04)	0.18 (0.01, 0.13)	<0.001
WBCs count, 10^9^/L	12.3 (7.6, 14)	11.5 (7.5, 13.4)	16.8 (9.9, 17.4)	<0.001
Platelet count, 10^9^/L	237 (159, 284)	238 (161, 281)	230 (141, 295)	0.36
Hemoglobin, g/dL	10.8 (9.3, 12)	10.8 (9.3, 12)	10.8 (9.8, 11.7)	0.59
Creatinine, mg/dL	3.59 (0.7, 1.3)	3.9 (0.7, 1.2)	1.47 (0.8, 1.7)	0.02
BUN, mg/dl	23.48 (16, 39)	21.9 (12, 26)	32.3 (17, 46)	<0.001
K^+^, mEq/L	4.07 (3.7, 4.6)	4.04 (3.7, 4.3)	4.23 (3.7, 4.6)	0.35
Na^+^, mEq/L	138.9 (137, 141)	138.9 (137, 141)	138.8 (136, 142)	0.95
PO_2_, mmHg	128 (78, 148)	127 (78, 147)	132 (71, 165)	0.94
PCO_2_, mmHg	42.3 (35, 47)	42.9 (36, 47)	41.2 (33, 48)	0.11
Length of ICU stay (days)	5.5 (1.7, 6)	5.4 (1.7, 5.6)	6.0 (1.9, 7.4)	0.04
Length of hospital stay (days)	14.2 (5.9, 16.8)	14.7 (6.2, 18)	11.39 (3.7, 13.6)	0.002

**Notes:** Data are expressed as mean value ± standard deviation, median (25th, 75th percentiles) or *n* (%). Kruskal–Wallis or Chi-square (or Fisher’s exact) tests were used for comparisons between groups. *Statistical significance (*P* < 0.05).

**Abbreviations:** ICU, intensive care unit; SOFA, sequential organ failure assessment data; APACHE III, acute physiologic and chronic health evaluation scoring system III; cTnT, cardiac troponin T; SIRS, systemic inflammatory response syndrome; K, serum potassium levels; Na, serum sodium levels; SBP, systolic blood pressure; DBP, diastolic blood pressure; Temp, temperature; SpO_2_, oxygen saturation; WBC, white blood cell; BUN, blood urea nitrogen levels; PCO_2_, partial pressure of carbon dioxide; PO_2_, partial pressure of oxygen; DVT, deep vein thrombosis; GCS, Glasgowcomascore; COPD, chronic obstructive pulmonary disease; NIV, non-invasive mechanical.

**Table 2:** Multivariable logistic regression analysis of factors associated with in-hospital mortality in critically ill patients with acute PE

Variables	OR	95% CI	*P*-value
cTnT	1.96	1.18–3.24	0.008
APACHE III	1.03	1.02–1.05	<0.001
Age	1.04	1.02–1.07	<0.001
BMI	0.94	0.88–0.99	0.032
Hemoglobin	1.21	1.03–1.42	0.016
WBC count	1.02	1.01–1.03	0.026
Invasive MV	3.11	1.69–5.72	<0.001
Malignant tumor	1.99	1.07–3.68	0.028

**Abbreviations:** OR, odds ratio; CI, confidence interval; APACHE III, acute physiologic and chronic health evaluation scoring system III; cTnT, cardiac troponin T; WBC, white blood cell; MV, mechanical ventilation.

## 3 Results

### 3.1 Baseline characteristics of the study cohort

Seventy one out of the 501 patients who were included initially died, thereby indicating 14.17% mortality. Finally, a total of 430 patients (average patient age: 65.6 ± 16.3 years) were included. The comparison of demographics, clinical features, and laboratory measurements between the two groups is presented in Table 1. A large proportion of patients who did not survive required invasive MV. No significant difference was noted in the prevalence of comorbidities between these two patient groups. The notable exception was the presence of malignant tumor (24.65% vs 36.6%, *P* = 0.03). The level of cTnT and APACHE III score in the patients who did not survive were significantly higher than those who survived (*P* < 0.05).

### 3.2 Association between factors and study outcome

The multivariate logistic regression analysis indicated a positive association of cTnT and APACHE III score with in-hospital mortality in patients with PE. The odds ratio of cTnT and APACHE III score for in-hospital mortality was 1.96 (95% confidence interval [CI] = 1.18–3.24, *P* = 0.008) and 1.03 (95% CI = 1.02–1.05, *P* < 0.001), respectively (Table 2).

### 3.3 ROC curve of cTnT

**Figure 2:**
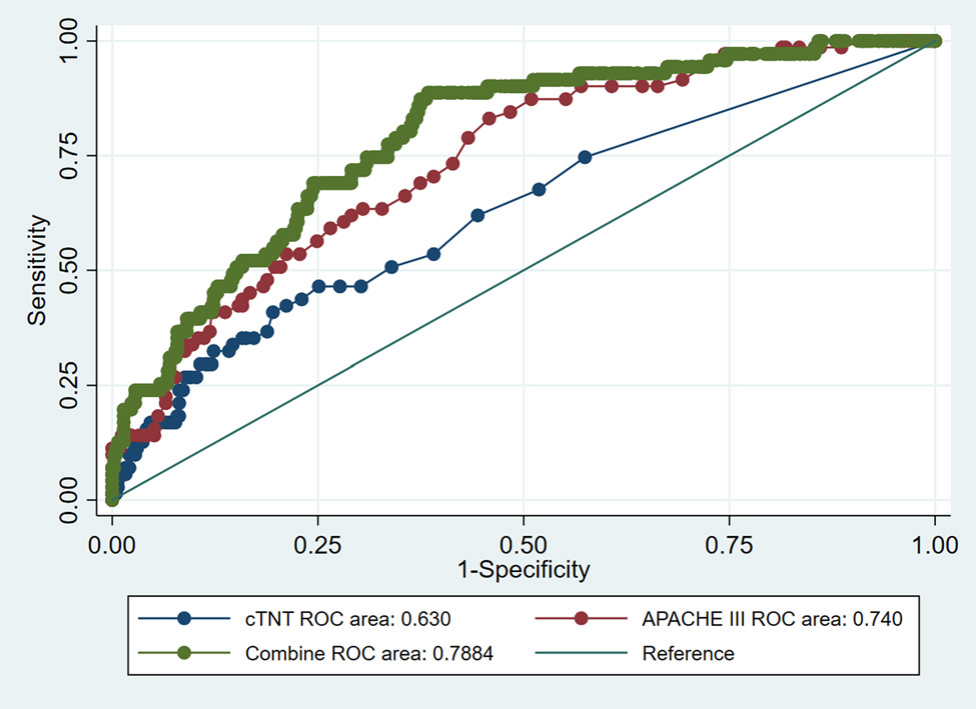
The area under the ROC curve (AUC) of cTnT combined with the APACHE III score was the highest (0.788), followed by that of the APACHE III score and cTnT alone (0.740 and 0.630, respectively). All *P* values are less than 0.05. Abbreviations: APACHE III, acute physiologic and chronic health evaluation scoring system III; cTnT, cardiac troponin T.

The AUC of the cTnT level and APACHE III score for in-hospital mortality in patients with PE was 0.630 (95% CI = 0.586–0.672, *P* = 0.03) and 0.740 (95% CI = 0.699–0.778, *P* = 0.02), respectively. The discriminatory cTnT and APACHE III threshold values for in-hospital mortality were 0.08 ng/L and 38, respectively. The sensitivities for the same were 46.48 and 83.10%, respectively, and the specificities were 74.88 and 54.19%, respectively. The cTnT and APACHE III were combined in the logistic analysis model, and a regression equation was derived to calculate in-hospital mortality. Consequently, the AUC was found to increase to 0.788 (95% CI = 0.734–0.840, *P* = 0.025), whereas the sensitivity and specificity were found to increase to 84.5 and 71.4%, respectively (Figure 2; Table 3). The discriminatory cTnT and APACHE III threshold values were the same cut-off (0.08 ng/L and 38, respectively) for the combined logistic regression analysis.

**Table 3:** Predictive value of troponin T, APACHE III, and the combination thereof for in-hospital mortality in critically ill patients with acute PE

	Troponin T	APACHE III	Troponin T + APACHE III
Optimal threshold	0.08	38	cTnT 0.08
			APACHE III 38
Sensitivity	46.48	83.10	84.5
Specificity	74.88	54.19	71.4
**+**LR	1.85	1.81	2.5
-LR	0.71	0.31	0.18
AUC	0.630	0.740	0.788
95% CI	0.586–0.672	0.699–0.778	0.734–0.845

**Abbreviations:** AUC, area under the receiver operator characteristic curve; CI, confidence interval. +LR, positive likelihood ratio; -LR, negative likelihood ratio.

## 4 Discussion

As a marker of cardiac disease, cTnT is particularly specific and sensitive for myocardial infarction. Four types of troponin, namely HsTnT, HsTnI, cTn, and cTnI, are widely used in clinic. All these troponins are associated with cardiovascular disease mortality and heart failure. El-Menyar et al. demonstrated that regardless of the measurement methods and troponin types, an elevated troponin level was significantly associated with high mortality in patients with PE [11]. Welsh et al. observed that cardiac troponin I is highly specific to the mortality risk in composite coronary disease and cardiovascular disease, whereas cTnT is closely related to mortality in non-cardiovascular disease [12]. Compared with other troponin types, cTnT has been most widely studied as an in-hospital mortality predictor [13,14,15]. Most studies have evaluated patients in the general ward or outpatient department, and this study is the first to report an association of cTnT with acute PE in patients in the ICU. The multivariable logistic regression analysis in the present study exhibited that an elevated cTnT level is associated with in-hospital deaths in patients with PE in the ICU. In the ROC curve analysis, the specificity of cTnT for in-hospital mortality prediction was found to be 74.88, with an AUC of 0.630. A correlation between these parameters and in-hospital mortality was noted, which is consistent with the findings of other studies.

The threshold troponin value in all-cause in-hospital mortality prediction has been controversial due to different troponin types and measurement methods, which contribute to a large difference between the values. Moreover, immeasurable extreme values such as >25 or <0.01 ng/L are often observed. Thus, several studies have considered certain extreme values such as 0.01 µg/L [16], 0.03 ng/mL [14], and 0.1 µg/L [17] as the cut-off to evaluate mortality in patients with PE. Because these extreme values account for a small proportion, we reassigned them. The cTnT values <0.01 ng/L were considered to be 0.009 ng/L, whereas those >25 ng/L were considered to be 25.1 ng/L. A novel discriminatory threshold value of 0.08 ng/L was observed in the ROC analysis.

A few studies have evaluated the PE scoring systems. The sPESI is a widely used tool for determining the severity and prognosis of PE. Recently, the modified Glasgow prognostic score was also reported useful for predicting in-hospital mortality in patients with stable hemodynamics [18]. However, both these methods are unsuitable for assessing mortality in critically ill patients with acute PE. Several scoring systems such as the SOFA, APACHE score, SIRS, Glasgow score, and SAPS have been developed for the assessment of critically ill patients. All these systems were developed for assessment in different groups of patients in the ICU; however, their precision in patient subgroups is poor [19]. Patients with PE in the ICU are typically evaluated using the commonly used scoring systems. The present study exhibited that not all scoring systems can predict mortality. The APACHE III scoring system proved to be the only acceptable prognostic scoring system, with a discriminatory threshold value for in-hospital mortality of 38. However, the specificity was only 54.19%. To further improve the in-hospital mortality prediction of patients with acute PE, we investigated whether a combination of cTnT and APACHE III score could improve sensitivity and specificity of in-hospital mortality prediction. The present study demonstrated that a combination of cTnT and APACHE III score could increase the AUC to 0.788 and the sensitivity and specificity to 84.5 and 71.4%, respectively. The area under the ROC of combining cardiac color ultrasound and cTnT to predict in-hospital mortality was >0.9. Studies have suggested that color Doppler echocardiography can predict patient prognosis by monitoring the right ventricular function [20]. The APACHE III score measurement is far easier to perform than the cardiac color ultrasound, particularly in patients with critical illness. The present study exhibited that a combination of cTnT and APACHE III score can significantly improve in-hospital mortality prediction. This finding could be helpful for clinicians in charge of patients and for managers involved in resource allocation and performance evaluation.

## 5 Limitations of the study

This study has certain limitations. We did not include the treatment type into the multivariate analysis, because most patients included in this study had received standardized treatment during ICU stay, suggesting that the type of treatment had a slight effect on the results. Additionally, only a few patients such as those with massive hemorrhage, gastrointestinal hemorrhage, or intracranial hemorrhage were unable to receive standardized treatment, and such patients could not be identified by us during the selection process. However, we propose a new method to predict the prognosis of critically ill patients with acute PE.

## 6 Conclusion

cTnT and APACHE III scores are significantly associated with all-cause in-hospital mortality in critically ill PE patients. Combination of cTnT and APACHE III score can significantly improve in-hospital mortality prediction.
